# Mixed Matrix Membranes Based on Torlon^®^ and ZIF-8 for High-Temperature, Size-Selective Gas Separations

**DOI:** 10.3390/membranes11120982

**Published:** 2021-12-15

**Authors:** Matilde De Pascale, Francesco Maria Benedetti, Elsa Lasseuguette, Maria-Chiara Ferrari, Kseniya Papchenko, Micaela Degli Esposti, Paola Fabbri, Maria Grazia De Angelis

**Affiliations:** 1 Department of Civil, Chemical, Environmental and Materials Engineering, University of Bologna, 40131 Bologna, Italy; matilde.depascale@gvs.it (M.D.P.); fmben@mit.edu (F.M.B.); K.Papchenko@sms.ed.ac.uk (K.P.); micaela.degliesposti@unibo.it (M.D.E.); p.fabbri@unibo.it (P.F.); 2 GVS S.p.A via Guido Rossa 30, 40069 Zola Predosa, Italy; 3 Osmoses Inc., 444 Somerville Ave, Somerville, MA 02143, USA; 4 School of Engineering, University of Edinburgh, Sanderson Building, Robert Stevenson Road, Edinburgh EH9 3FB, Scotland, UK; E.Lasseuguette@ed.ac.uk (E.L.); M.Ferrari@ed.ac.uk (M.-C.F.); 5 Italian Consortium for Science and Technology of Materials (INSTM), 50121 Firenze, Italy

**Keywords:** mixed matrix membranes, CO_2_ capture, gas separation

## Abstract

Torlon^®^ is a thermally and plasticization-resistant polyamide imide characterized by low gas permeability at room temperature. In this work, we aimed at improving the polymer performance in the thermally-enhanced He/CO_2_ and H_2_/CO_2_ separations, by compounding Torlon^®^ with a highly permeable filler, ZIF-8, to fabricate Mixed Matrix Membranes (MMMs). The effect of filler loading, gas size, and temperature on the MMMs permeability, diffusivity, and selectivity was investigated. The He permeability increased by a factor of 3, while the He/CO_2_ selectivity decreased by a factor of 2, when adding 25 wt % of ZIF-8 at 65 °C to Torlon^®^; similar trends were observed for the case of H_2_. The MMMs permeability and size-selectivity were both enhanced by temperature. The behavior of MMMs is intermediate between the pure polymer and pure filler ones, and can be described with models for composites, indicating that such materials have a good polymer/filler adhesion and their performance could be tailored by acting on the formulation. The behavior observed is in line with previous investigations on MMMs based on glassy polymers and ZIF-8, in similar conditions, and indicates that ZIF-8 can be used as a polymer additive when the permeability is a controlling aspect, with a proper choice of loading and operative temperature.

## 1. Introduction

Membrane materials with good size selectivity and thermal resistance are required to remove carbon dioxide from hydrogen or helium; indeed, the separation performance is enhanced, both in terms of permeability and selectivity, at high temperatures [1,2,3,4,5]. This aspect can be exploited in processes where hydrogen is produced through gasification or steam reforming at a rather high temperature, avoiding the need to cool down the gas stream for the separation stage, which burdens the process with a significant energy penalty. Another application in which size-selective membranes are required is the removal of He from natural gas (mainly CH_4_), which requires a very high He/CH_4_ selectivity. Such process is not performed with membranes yet, due to the low partial pressure of Helium in natural gas streams, but will eventually become relevant as the price of Helium is expected to rise in the next years [6].

Unfortunately, polymeric membranes, which are the most economic selective materials for gas mixtures, are normally not endowed with a high thermal resistance compared to inorganic or metallic membranes. Furthermore, polymeric size-selective membranes obey a well-known tradeoff between the permeability, related to the productivity of the process, and the selectivity, determining the process efficiency [7,8]. It is not easy to enhance permeability and selectivity at the same time on the membrane material. Thus, there is an upper limit to the polymeric membranes performance. Several different strategies have been proposed by researchers to fabricate materials with improved performance [9,10,11,12,13]; among them, there is the dispersion of selective nanoporous particles into polymeric matrix. Such strategy has the aim of maintaining the processability and flexibility of the polymeric material, which constitutes the scaffold of the membrane, while improving the permeability and/or selectivity with the proper active additive. Those composite structures are usually called Mixed-Matrix Membranes (MMMs). A large number of fillers has been proposed and added to organic polymers in the literature, spanning from silica particles to zeolites, from graphene platelets to carbon molecular sieves (CMS), carbon nanotubes (CNTs), metal organic frameworks (MOFs), and covalent organic frameworks (COFs) [14,15,16,17,18,19,20,21,22,23].

MOFs were first introduced some years ago [24], and were immediately adopted in gas separation and storage due to their unique portfolio of properties and their structural tunability [25,26]. Soon after the introduction of the MOFs, membrane scientists started to develop MMMs based on organic polymers and MOF particles [27]. One of the key properties of MOFs in Mixed Matrix Membrane formation is their intrinsic affinity with organic polymer matrices, which makes them more suitable than zeolites as dispersed fillers. Indeed, a good affinity is a vital pre-requisite to ensure a good adhesion between the two phases and avoid the formation of voids at the interface, which can compromise selectivity and stability [28,29]. On the opposite side, it is important to avoid interpenetration between the two phases which could reduce the surface available for gas separation [30]. Besides their good intrinsic affinity with organic matrices, several strategies have been proposed to further improve the compatibility of MOF-based MMMs [31,32,33].

Within the MOF family, ZIFs (Zeolitic Imidazole Frameworks) are particularly interesting as they are isomorphic with zeolites [34]. In particular, the inorganic aluminum–silicate structure is replaced by organic imidazole linkers coordinated with metal ions into regular frameworks. ZIFs have a monomodal pore size distribution, which is particularly favorable to the separation of small gas molecules [35,36]. However, the theoretical separation factor, obtained based on the known crystallographic structure, is seldom reached experimentally due to the so-called “breathing” phenomenon, which causes the framework to fluctuate around its original configuration and the pores to broaden out [37,38]. Being part of the MOF family, ZIFs are available in a wide range of designs obtained by changing the organic linkers and the coordination metal [34]. This leads to different topologies and to different dimensions of the pores, ranging from 0.7 Å in the case of ZIF-61 to 13.1 Å in the case of ZIF-70.

With the purpose of using a commercial material available in the open market, we chose ZIF-8 as filler in this work. The pore diameter is reported to be 3.4 Å, between the effective diameter of small gases such as He (2.6 Å), H_2_ (2.90 Å) and CO_2_ (3.3 Å) and larger gases like N_2_ and CH_4_ (3.66 Å and 3.81 Å) [39]. As a consequence, ZIF-8 is H_2_- and He-selective over those gases and also has a moderate selectivity for the He/CO_2_ couple as well as for the H_2_/CO_2_ one [40,41,42,43,44]. Indeed, the He/CO_2_ selectivity has been reported to vary between 1.6 and 2.2 at room temperature for ceramic-supported polycrystalline ZIF-8 membranes and between 2.6 and 4.0 for single crystal ZIF-8 membranes [45]. Despite its larger kinetic diameter, the H_2_ permeability in ZIF-8 is higher than that of Helium and consequently, the H_2_/CO_2_ selectivity ranges between somewhat larger values, i.e., 3.9 and 4.6. However, some studies indicate that, due to its flexibility, ZIF-8 could be actually able to reach larger pore diameters and accommodate penetrants as large as 4.0–4.2 Å [46,47].

The polymer chosen as matrix is a polyamide imide, a high performance polymer with excellent thermal and plasticization resistance, although characterized by rather low fractional free volume and permeability [48,49,50,51], commercially sold as Torlon^®^. The polymer however shows high intrinsic size selectivity, based on large diffusion differences between gases of slightly different size [48,49,50,51], and, due to a high thermal resistance, can be used as membrane in relatively high temperature separations, such as those involving removal of CO_2_ from hydrogen [52,53]. Furthermore, it has been proven by several studies that such polymer can be obtained in the form of a hollow fiber membrane both in its pure form and in combination with other polymers [48,54,55,56]. Unfortunately, the excellent properties of this polymer cannot be exploited for gas separation at room temperature due to its low permeability, and for this reason, several attempts were made in the literature to modify it in order to enhance its permeation flux, such as the combination with other polymers of higher free volume [49,50,56,57].

In this work, we decided to follow a different approach, and in particular, we dispersed particles of the highly permeable ZIF-8 with the aim of improving the polymer permeability. ZIF-8 can be incorporated easily into organic polymers, compared with inorganic zeolites, and it was used for the preparation of several MMMs [58,59,60,61,62,63,64,65,66,67]. It was also proven that ZIF-8 has high hydrothermal stability and preserves its structure after exposure to N_2_ at 550 °C and immersion in boiling water for 7 d; that is attributed to its hydrophobicity [68], confirmed by water vapor sorption experiments [69] and molecular simulations [70]. ZIF-8 also has a high gas sorption capacity, as its BET surface area is reported to be 1630 m^2^/g [68]. The effect of the filler addition on the membrane selectivity depends on the initial polymer properties, as the size selectivity of ZIF-8 membranes towards He/CO_2_ and H_2_/CO_2_ mixtures is not high, ranging between 2 and 4, respectively [48,49,50,51].

A slight improvement of He/CO_2_ and H_2_/CO_2_ selectivity was observed after adding ZIF-8 to matrices with relatively low selectivity such as PPO [71], PSf [72], and PPEES [64], while a decrease of selectivity was observed after addition to the more selective matrices such as Matrimid^®^ [58,59] and PBI [60,61,62]. 

Previous studies have investigated the use of Torlon^®^ in membrane separation (CO_2_/CH_4_), as it was believed that this material, due to its tightly packed structure, could resist to the plasticization induced by high pressure CO_2_ [48,51]. Such tight packing is due, as demonstrated via IR spectroscopy, to the fact that almost all the N-H groups of the polymer are connected either through inter-chain or intra-chain hydrogen bonds [48]. Indeed, Kosuri and Koros [48] showed that hollow fibers of Torlon^®^ can be produced defect-free, and that during a pressurization cycle their CO_2_ permeance remains unvaried up to 75 atm. However, an hysteresis was observed in the depressurization cycle that is symptomatic of an induced swelling of the matrix, which requires some time to relax [48]. More recently, Forman et al. studied the self-diffusion of ethane and ethylene into Mixed Matrix Membranes formed by Torlon^®^ and ZIF-11, observing that the presence of Torlon^®^ acts as a physical constraint on ZIF-11 crystals, possibly reducing its flexibility and even its pore dimensions [73,74]. Indeed, the intracrystalline self-diffusion coefficient of the two vapors in the confined ZIF-11 is lower than the same value measured in a “free” bed of pure ZIF-11 crystals. The same effect is not observed in MMMs formed by ZIF-11 and other two polyimides, Matrimid^®^ and 6FDA-DAM: such interesting features of Torlon^®^ may be due to its higher bulk modulus with respect to those two polymers, and to a different adhesion degree with the filler [73,74]. Interestingly enough, the diffusion-selectivity of ZIF-11 was not affected by the presence of Torlon^®^. Yong et al. prepared membranes based on blends of Torlon^®^ and carboxylated PIM-1 (cPIM-1) to overcome the permeability limits of Torlon^®^, and the selectivity ones of cPIM-1, finding that the blends show a good intermediate behavior between the two polymers, with performances close to the upper bound for O_2_/N_2_, CO_2_/CH_4_, CO_2_/N_2_ and H_2_/N_2_ [50]. Dibrov et al. [54] fabricated Torlon^®^-based hollow fiber membranes to investigate their He/CH_4_ separation performance up to 80 bar. They found a Helium permeance of 100 L(STP)/m^2^ h bar and a He/CH_4_ selectivity of 340 [54].

In the present work, we fabricated thick, self-standing composite films based on Torlon^®^ with up to 25 wt % of ZIF-8. Permeation and diffusion of He, H_2_, and CO_2_ were investigated from 35 to 65 °C to assess the thermal dependence of the transport properties. Membranes were also characterized by SEM analysis to support the interpretation of transport data in correlation with the structural features. The data were compared to previous ones obtained on similar Mixed Matrix Membranes, and compared to the Maxwell–Wagner–Sillars model.

## 2. Materials and Methods

### 2.1. Polymer, Filler

**Figure 1:**
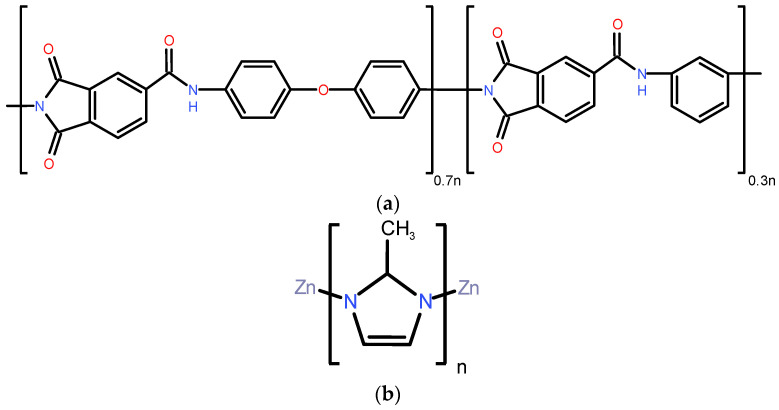
(**a**) Torlon^®^ and (**b**) ZIF-8 framework chemical structures. Both pictures are from this work.

The typical structure of the polymer (Torlon^®^ 4000 TF polyamide imide) used for the fabrication of the mixed matrix membranes is shown in Figure 1a.

Torlon^®^ is a resin commercially used as a coating material or additive, and it is specifically introduced when chemical resistance, thermal stability, and mechanical durability are needed. It is soluble in dipolar aprotic solvents such as N-methyl pyrrolidone (NMP), dimethylacetamide (DMAC), dimethyl sulfoxide (DMSO), and dimethyl formamide (DMF). The materials used in this work is Torlon^®^ 4000 TF and was kindly provided in powder form by Solvay Specialty Polymers. Polyamide imides have not been investigated as deeply as polyimides in membrane technology, primarily due to their lower permeability due to the presence of the amide group.

Some relevant physical properties of this material are summarized in Table 1.

The commercial sieve selected to produce our MMMs is ZIF-8 (Basolite^®^ Z1200, Cat. 691348 produced by BASF, Ludwigshafen am Rhein, Germany), whose structure is reported in Figure 1 [30,34,40,68]. The main properties are listed in Table 2. The manufacturer provided data on D50 of 4.90 µm. No further grinding was performed on the fillers used to produce MMMs to avoid damaging the crystals structure.

### 2.2. Membranes Preparation

Torlon^®^ in powder form was dried overnight at 120 °C under vacuum and then mixed to NMP in a 3 wt % solution. The mixture was sonicated for 30 min and then stirred until dissolution of the polymer. ZIF-8 was subsequently added to the solution at 6, 15, and 25 wt % with respect to the polymer, after activating the crystals at 200 °C under vacuum overnight. The polymer–filler solution was stirred for 2 days at room temperature and poured onto a glass Petri dish. This concentration of solution reduces the deposition and the aggregation of particles during the casting step [75]. The Petri dish was then placed on a hot plate under the fume hood overnight to let the membrane cast and to quicken evaporation of the solvent. The casting temperature was set to 70 °C since, after several attempts, it was found to be the most suitable one to obtain a defect-free film with a good particle dispersion. To avoid turbulences inside the fume hood and, therefore, ripples on the surface of the membranes, a constant air flow in the casting environment was set. Before testing, the membranes were dried in the oven at 200 °C under vacuum overnight for the complete evaporation of the residual solvent. Neat polymer films were also prepared for comparison reasons.

### 2.3. Morphological Characterization

The morphology of ZIF-8/Torlon^®^ MMMs was investigated at different loadings of the MOF by means of scanning electron microscopy (SEM). The membranes have been examined with Carl Zeiss SIGMA HD VP Field Emission operating at 15 kV. Before SEM analysis, the samples were fractured in liquid nitrogen and then sputtered with a layer of 9 nm gold to form a conductive surface.

### 2.4. Thermal Properties

The thermal behavior of the prepared films was evaluated by differential scanning calorimetry (DSC, Q10, TA Instruments), fitted with a standard DSC cell and equipped with a Discovery Refrigerated Cooling System (RCS90, TA Instruments, Waters Corporation, Milan, Italy). The system was calibrated both in temperature and enthalpy with Indium standard. Measurements were performed under dry nitrogen flow at 50 mL⋅min^−1^ on samples of approximately 15 mg placed in an aluminum pan. Samples were subjected to a heating cycle from +25 to +350 °C (+300 °C for neat Torlon^®^) with a heating rate of 10 °C⋅min^−1^. DSC curves were processed with TA Universal Analysis 2000 software (TA Instruments) in order to extrapolate the glass transition temperature (T_g_).

### 2.5. Gas Permeation Experiments

The permeability of He, H_2_, and CO_2_ was evaluated at different temperatures (35 and 65 °C). All gases were purchased from S.I.A.D. Spa (Bergamo, Italy) with purity at or above 99.99% and used as received. The experiments were carried out first on helium, hydrogen, and then on CO_2_, to prevent any conditioning effect of the sample [76,77,78]. Each permeability experiment was performed at an absolute upstream pressure of 1.3 bar. To investigate the effect of the temperature at different loadings of the sieve, the permeability was evaluated at 35 °C and at 65 °C on the same sample. The fixed-volume, variable-pressure manometric technique previously described elsewhere [79] was implemented to perform the experiments.

The permeability 𝒫 can be evaluated from such an experiment at steady state by the following equation:

(1) $$𝒫=\frac{V_{d}}{RT}\frac{l}{A}\frac{1}{\left({\bar{p}}_{u}-{\bar{p}}_{d}\right)}{\left(\frac{dp_{d}}{dt}\right)}_{SS}$$

in which R is the gas constant, T is the temperature, l the membrane thickness, $V_{d}\;$the downstream volume, A the membrane area, and ${\bar{p}}_{u}$ and ${\bar{p}}_{d}$ the time-averaged upstream and downstream gas pressure respectively. ${\left(\frac{dp_{d}}{dt}\right)}_{SS}$ is the time rate of change of instantaneous downstream pressure at steady state (*SS*). The uncertainty on the permeability values was calculated by considering the experimental error made to measure the various quantities involved in its calculation by the propagation of error approach [80].

The ideal selectivity between gas *A* and *B*, $\alpha_{A/B}$, can be calculated for each gas pair as follows, valid when the downstream pressure is negligible with respect to the upstream one as in the present case:

(2) $$\alpha_{A/B}=\frac{y_{A,d}/y_{B,d}}{y_{A,u}/y_{B,u}}≅\frac{{𝒫}_{A}}{{𝒫}_{B}}=\frac{D_{A}}{D_{B}}\frac{S_{A}}{S_{B}}=\alpha_{A/B}^{D}\alpha_{A/B}^{S}$$

$y_{A,d}$ and $y_{B,d}$ are the molar fraction in the downstream side of the membrane of gas *A* and *B* respectively, while $y_{A,u}$ and $y_{B,u}$ are those in the upstream side of the film. ${𝒫}_{A}$ and ${𝒫}_{B}$ are the permeability of gas *A* and *B* respectively. If the solution-diffusion model holds true, the permeability can be seen as the product of the diffusivity D and the solubility coefficients S, while the ideal selectivity can be split into the product of diffusivity–selectivity $\alpha_{A/B}^{D}$, and solubility–selectivity$\;\alpha_{A/B}^{S}$.

The diffusivity has been calculated with the time-lag, $\theta_{L}$, that is the intercept on the time axis of the linear curve representing the steady state increase of downstream pressure with time. The time-lag is a measure of the characteristic time required to the gas molecule to dissolve in the polymer matrix and to diffuse through the film, and for a zero initial concentration of gas across the membrane, it is related to the diffusivity through the following equation [81,82]:

(3) $$D=\frac{l^{2}}{6\theta_{L}}$$

## 3. Results

### 3.1. Membranes

The MMMs obtained, depicted in Figure S1, are characterized by the typical yellow translucent aspect which becomes more opaque when the filler content is increased. All membranes show no visible aggregates and have good flexibility. In Table S1, we reported a list of the samples fabricated and of the various tests performed on each one of them, together with their thickness.

### 3.2. SEM Analysis

**Figure 2:**
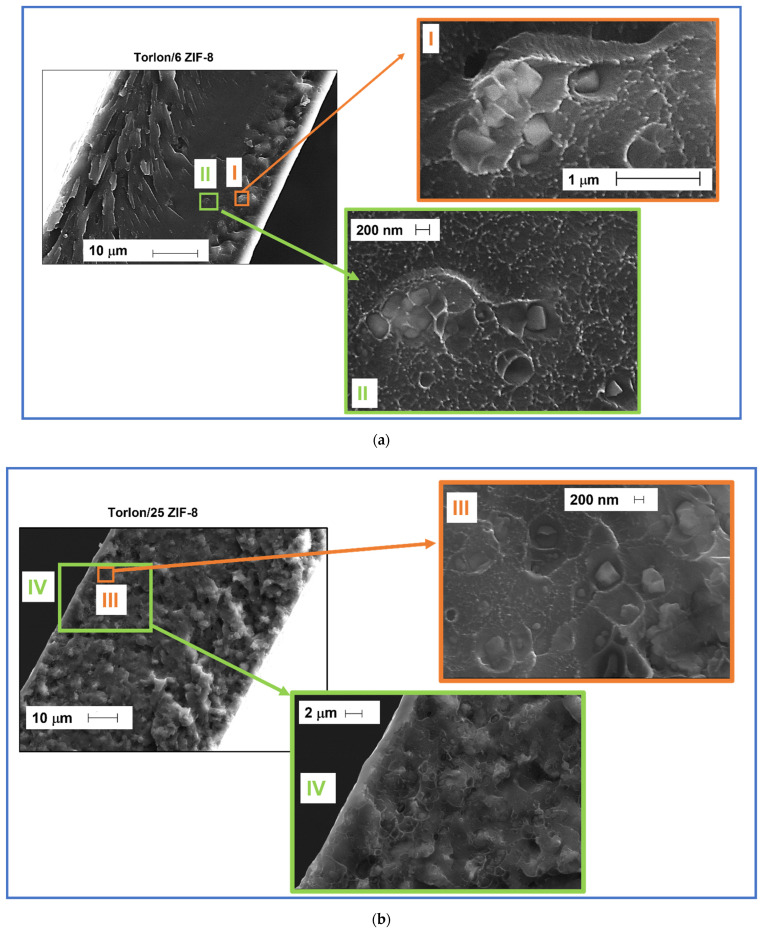
SEM images of (**a**) Torlon/6 ZIF-8 and enlarged view of two different areas (frames I and II); (**b**) Torlon/25 ZIF-8: high resolution view of smaller particles (frame III); low resolution view of larger aggregates (frame IV).

SEM images of the cross section of pure Torlon^®^ membrane and Torlon/ZIF-8 MMMs are shown in Figure S2, where we can detect the effect of the filler addition on the membrane cross-section. In particular, the smooth and compact section of the pure polymer becomes increasingly disrupted by the addition of filler. The formation of wrinkles and roughness seems to be associated to the presence of particle aggregates that deform the surface of the membrane cross section. In Figure 2, we report details of the ZIF-8 particles embedded in the MMMS considered. In Figure 2a, which shows the membrane Torlon/6 ZIF-8, we can see details of micrometric ZIF-8 crystal aggregates, with size compatible to that declared by the producer, embedded in the membrane. In Figure 2b, showing Torlon/25 ZIF-8 sample details, we can see single isolated crystals (Frame III) together with larger aggregates with a marked interconnection (Frame IV).

### 3.3. Thermal Behaviour

The thermal behaviour of the neat and composite materials containing 6 and 15 wt % of ZIF-8 investigated by DSC as a function of the filler content is summarized in Table 3, while the thermal scans are reported in Figure S3 in the Supplementary Information. The glass transition of Torlon^®^ is observed at 267 °C, which is in good agreement with literature value [50]. The T_g_ value increases by 30 °C (297 °C, +5%) after the addition of 6 wt % of ZIF-8, suggesting that the mobility of the macromolecular chains is reduced when filler particles are introduced between the polymer chains. The further incorporation of a bigger amount of filler (15 wt % of loading) does not have any effect on the T_g_ value (296 °C), since no additional reduction in the mobility is induced.

Variations of the polymer glass transition temperature due to the incorporation of the filler were observed also for other ZIF-8 containing MMMs depending on the filler loading, the chemical structure of the polymer, and on the polymer–filler interactions and hindering ability. In ZIF-8 incorporating PPO-based membranes [71], the increase of T_g_ was linear with filler loading and limited to within 1.5% at the highest loading inspected (45%). This suggests that the filler particles have limited hindering ability on the polymer chains segmental motion even for high loads, leading to a small T_g_ variation. Song and co-workers [59] investigated the glass transition of ZIF-8/Matrimid^®^ composite membranes. In their work, T_g_ increases of 6% when 30 wt % of filler was added indicating that the molecular mobility of Matrimid^®^ is more affected by the presence of ZIF-8 particles compared to PPO. A different behavior was observed in ZIF-8/PPEES systems reported by Diíaz et al. [64]. It was found that the T_g_ of the MMMs is independent on the filler content suggesting that strong polymer–filler interactions are absent in such composite membranes.

Moreover, as reported in Figure S3, no peaks that can be ascribed to decomposition phenomena are depicted in DSC thermograms for neat Torlon^®^ and composite membranes, confirming the already well known high thermal stability of the used materials [68,71]. In particular, at the tested permeation temperatures of 35 and 65 °C, but also at higher temperatures (up to and above 300 °C, for neat Torlon^®^ and composite membranes, respectively), no degradation peaks are observed for all the tested compositions, revealing that the addition of increasing amounts of ZIF-8 does not induce deterioration phenomena.

### 3.4. Permeability and Selectivity

#### 3.4.1. Data on Pure Torlon^®^ and Comparison with the Literature

**Figure 3:**
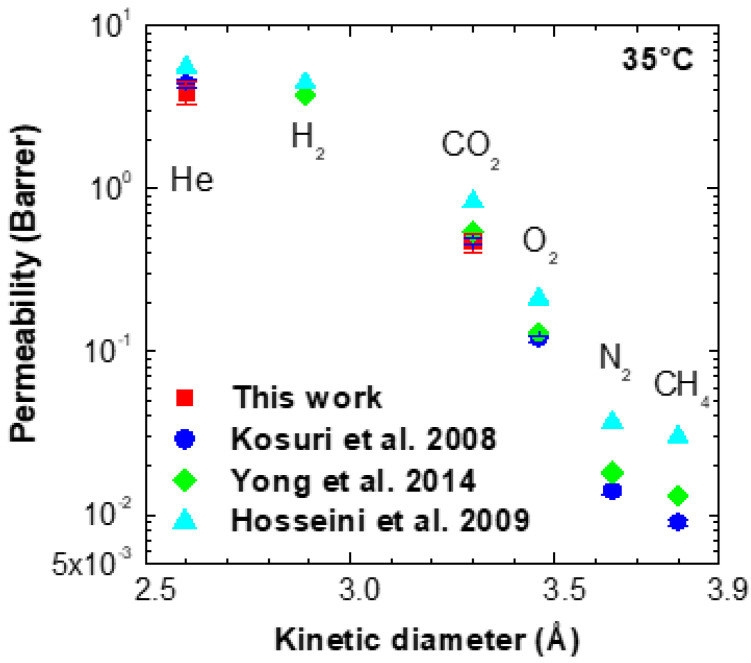
Gas permeability in Torlon^®^ from this work and literature works [48,49,50].

Permeability data on pure Torlon^®^ at room temperature are reported in Figure 3 and Table S2 as a function of the gas kinetic diameter and compared with literature ones. In this work, tests were carried out with He and CO_2_ and an estimate for H_2_ was also provided, as discussed in the following. The marked size-sieving nature of the polymer, with permeability decreasing by more than two orders of magnitude going from the smaller gas (He, kinetic diameter = 2.6 Å) to the larger one (methane, kinetic diameter = 3.8 Å) is apparent from the data reported in Figure 3. The data on He and CO_2_ in pure Torlon^®^ are consistent with the literature, especially considering the general variability of the results. In particular, at 35 °C, our CO_2_ permeability value is equal to 0.47 ± 0.06 Barrer, while the He permeability is 3.9 ± 0.6 Barrer.

Based on the data in Table S2, the ideal selectivity for the He/CO_2_ mixture for pure Torlon^®^ at 35 °C lies between 6.7 and 9.4, while it is somewhat lower, varying from 5.3 to 6.9, for the H_2_/CO_2_ couple, in agreement with the lower size of Helium with respect to Hydrogen. Indeed, according to Hosseini et al. [49], the He/H_2_ permeability ratio for pure Torlon^®^ equals 1.26 at 35 °C.

#### 3.4.2. Permeability and Ideal Selectivity

In this section, we report permeability data as a function of gas type, temperature, and filler content in the various matrices. As mentioned above, one of the main targets of this work was to address the H_2_/CO_2_ separation. For safety issues, the handling of Hydrogen in our lab is limited and we carried out only an essential minimum set of data, using careful extrapolations to estimate its behavior in other systems.

**Figure 4:**
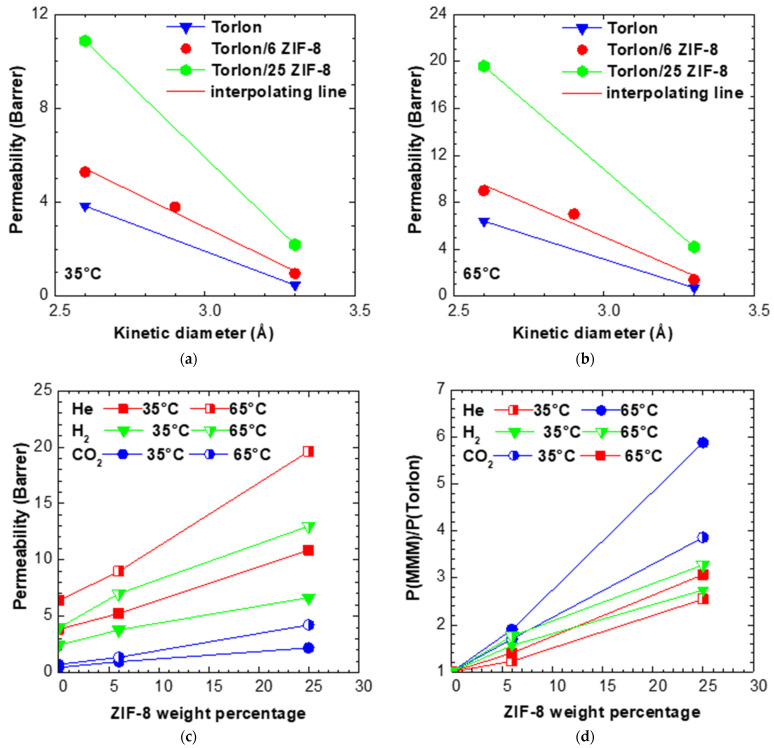
Permeability in Torlon/ZIF-8 MMMs as a function of gas kinetic diameter at (**a**) 35 and (**b**) 65 °C. Interpolating line parameters for the Torlon/6 ZIF-8 are reported in Table 4. (**c**) Permeability and (**d**) relative permeability increase of various gases at 35 and 65 °C in Torlon/ZIF-8 MMMs, as a function of ZIF-8 weight percentage.

The data of permeability at 35 °C and 65 °C in the various mixed matrix membranes are reported in Table S3 and Figure 4a. In particular, Figure 4a,b show the permeability of the various gases in the different MMMS studied, as a function of the gas kinetic diameter. The tests on H_2_ were performed only on the matrix Torlon/6 ZIF-8: as it can be seen from the data relative to such membrane, there is a good linear relationship between the permeability and the gas kinetic diameter, at both temperatures inspected, as follows:

(4) 𝒫𝒫=−Aσ+B

The relationship has a high value of the correlation coefficient, R^2^, for both temperatures, as reported in Table 4. Moreover, the correlation is also followed, with very similar values of the coefficients **A** and **B**, also by the data measured for the three gases on pure Torlon^®^ in the literature [49]. The coefficients A and B are reported in Table 4.

**Table 4:** Parameters for the correlation represented by Equation (4).

	A (Barrer/Å)	B (Barrer)	R^2^
Torlon/6 ZIF-8, 35 °C	6.23	21.6	0.991
Torlon/6 ZIF-8, 65 °C	11.0	38.2	0.966
Torlon, 35 °C [49]	6.85	23.7	0.960

We can thus assume that linearity holds true between permeability and gas kinetic diameter in the limited range encompassing He, H_2_, and CO_2_ for all the membranes studied in this work, with the slope of the linear correlation allowed to vary for each sample. Such approximation has been used to estimate the permeability of H_2_ in samples of Torlon and Torlon/25 ZIF-8. The experimental and interpolated values of permeability for H_2_ in the different materials are reported in Table S3. The data of permeability at 35 and 65 °C as a function of filler content, are also reported in Figure 4c,d in terms of absolute permeability and relative permeability increase, respectively. From Figure 4c,d, one notices that the permeability values of He and CO_2_ are both enhanced by the presence of ZIF-8, because this filler has an intrinsically higher permeability for these gases with respect to pure Torlon^®^. The Helium permeability reaches values of about 10 and 20 Barrer at 35 and 65 °C, respectively, as can be seen in Table S3. The permeability towards CO_2_ also increases, and to a greater extent than He on a relative basis. In particular, at 35 °C the permeability of CO_2_ is enhanced by a factor of almost 4 at the highest ZIF-8 content, while that of Helium increases by 2.5 times. At 65 °C, such values increase, reaching a maximum permeability enhancement factor of almost 5 for CO_2_, and of almost 3 for Helium.

**Figure 5:**
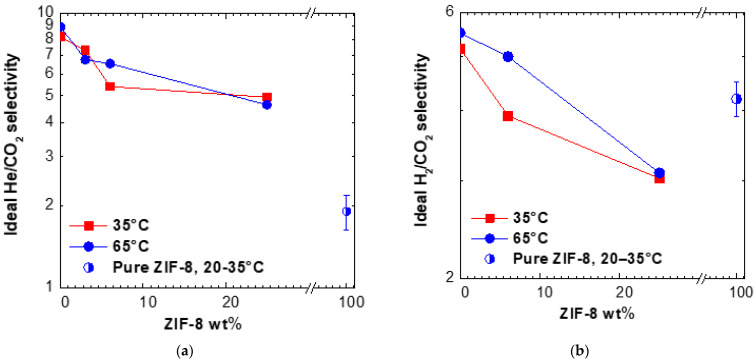
Ideal selectivity for (**a**) He/CO_2_ and (**b**) H_2_/CO_2_ at 35 °C and 65 °C in Torlon/ZIF-8 MMMs. Data for pure ZIF-8 from the literature (see Table S4).

The gas-dependent enhancement of permeability affects the ideal selectivity values reported in Figure 5a and Table S4. At both temperatures inspected, the He/CO_2_ selectivity decreases from a factor of about 8 for Torlon to around 5 for Torlon/25 ZIF-8. This behavior is due to the relatively large pore size of the filler, bearing an intrinsically lower size selectivity with respect to the neat polymer. However, the data are intermediate between the pure polymer and pure filler values, indicating that the MMMs do not show additional voids, which can sometimes form at the polymer–filler interface and cause a marked drop of size-selectivity.

The estimated H_2_/CO_2_ selectivity of Torlon (5.22 at 35 °C) also decreases after addition of ZIF-8, down to 3.0 for the matrix containing the highest loading, as reported in Figure 5b. The trend is qualitatively in line with the fact that ZIF-8 has a reported selectivity lower than Torlon^®^ for this gas couple. For Torlon/25 ZIF-8, the selectivity is below the value of pure ZIF-8: the reason for this trend is not clear, but may also be an artefact due to the uncertainty in the estimate of H_2_ permeability for such sample.

For both He/CO_2_ and H_2_/CO_2_ mixtures, the ideal perm-selectivity is mostly enhanced by temperature, as the permeability of CO_2_ is less enhanced by temperature with respect to that of smaller gases (He, H_2_). Such aspect is discussed in more detail in the following section about diffusivity.

#### 3.4.3. Effect of Filler Loading on Diffusivity and Diffusivity–Selectivity

**Figure 6:**
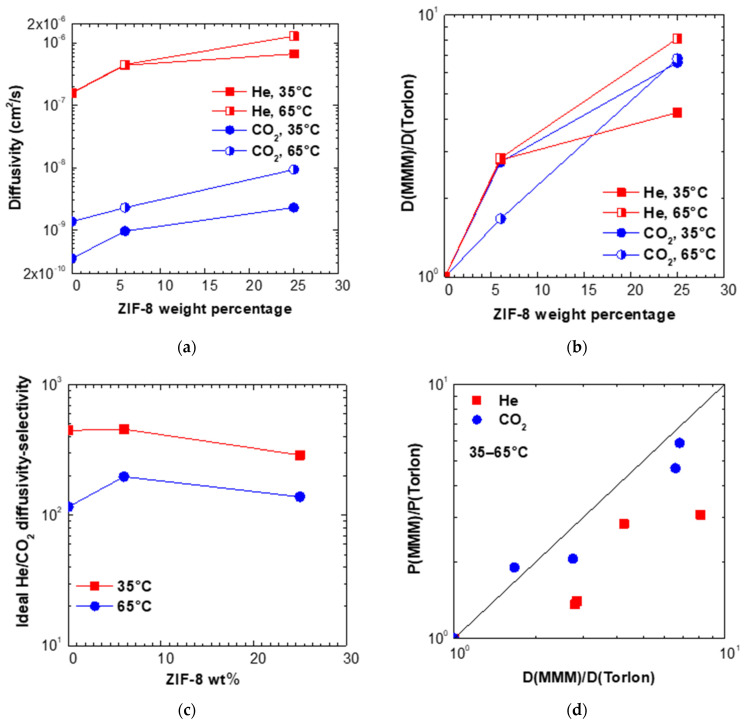
(**a**) Diffusivity and (**b**) relative diffusivity increase of various gases at 35 and 65 °C in Torlon^®^/ZIF-8 MMMs. (**c**) Ideal diffusivity–selectivity in Torlon/ZIF-8 MMMs. (**d**) Correlation between relative permeability variation and relative diffusivity variation after addition of various loadings of ZIF-8.

The diffusivity values in the various mixed matrices inspected as a function of filler content are reported in Table S5 and Figure 6a at 35 and 65 °C. On the other hand, in Figure 6b, we display the relative diffusivity enhancement at both temperatures versus filler weight fraction. As it can be noticed, the diffusivity increases for both gases after addition of ZIF-8, and the enhancement factors are comparable for the two gases. In particular, the maximum loading of ZIF-8 yields a relative increase of He diffusivity equal to 4.2 and 8.1 at 35 and 65 °C, respectively. The enhancement of CO_2_ diffusion coefficient at the highest loading of ZIF-8 seems a weaker function of temperature and is about 6.7 at both temperatures. The diffusivity selectivity trend, reported in Table S5 and Figure 6c, decreases with temperature, indicating that high temperatures are detrimental to the diffusion-based separation. This is somehow in contrast with the perm-selectivity behavior, which is enhanced by temperature in almost all matrices, as reported in Figure 5. Such result can be explained considering the different dependence on temperature of permeability and diffusivity for the two gases, He and CO_2_. The CO_2_ diffusivity is strongly enhanced by temperature, more markedly than Helium diffusivity, possibly because of its larger size. As a consequence, increasing the temperature reduces the He/CO_2_ diffusivity-selectivity.

For the permeability the situation is opposite, and this behavior can be explained considering the permeability as the product of solubility and diffusivity, two quantities which have opposite trends with temperature. Solubility is an exothermic process and is unfavored by temperature, while diffusion is a thermally enhanced process. Usually, the permeability follows the trend of diffusivity and increases with temperature, but such dependence is weaker for those gases, such as CO_2_, for which solubility forms an important contribution to permeation, and stronger for gases like He and H_2_ for which the solubility in the polymer is very small. Therefore, the temperature enhances more the permeability of He and H_2_ than that of CO_2_, and the He/CO_2_ perm-selectivity increases with temperature, which is promising for the high temperature membrane separation of these mixtures.

A direct comparison of the effect of filler addition on the diffusivity and permeability is reported in Figure 6d for the temperatures of 35 and 65 °C. This plot allows to compare the permeability enhancement brought about by the addition of filler to the diffusivity one, by comparing the data to the bisector. In most cases, the permeability variation is higher than the one experienced by diffusivity, especially for Helium. Following the solution-diffusion model, one would conclude that the amount of gas absorbed by the mixed matrix membrane is lowered by the presence of filler, leveraging somehow the diffusivity behavior. Such mechanism seems however not reasonable, because gas sorption levels in ZIF-8 are expected to be higher than those in a low free volume polymer such as Torlon^®^. Another possible explanation would be that such materials do not follow closely the solution-diffusion behavior, which is indeed strictly valid only for dense homogenous polymers. A separate analysis of the gas sorption in mixed matrix membranes based on ZIF-8 is currently under way to analyse this aspect in more detail, and will be the object of a future paper.

### 3.5. Comparison with Other Membranes Performances and with Robeson’s Upper Bound

**Figure 7:**
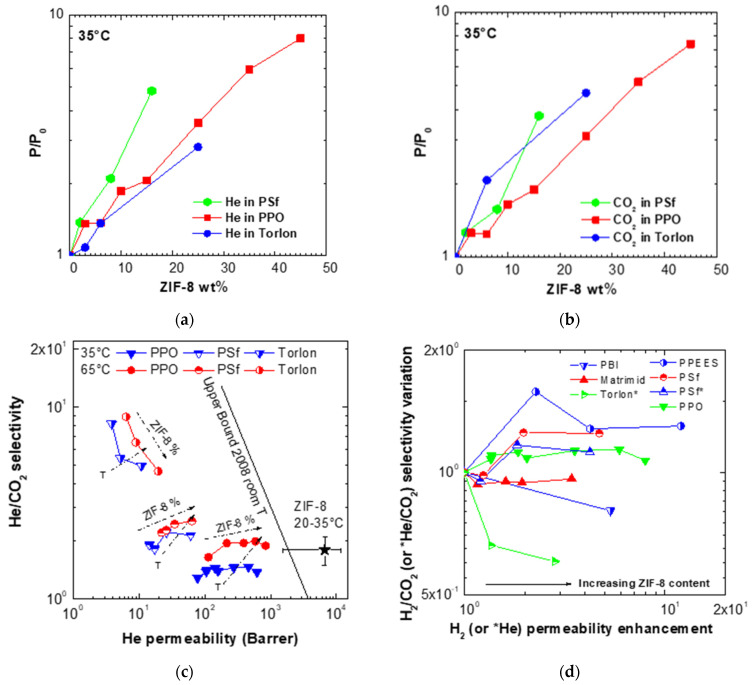
Relative (**a**) He and (**b**) CO_2_ permeability increase measured after addition of ZIF-8 to Torlon^®^ (this work), PSf [72], and PPO [71] at 35 °C. (**c**) Performance of mixed matrices based on ZIF-8 and Torlon^®^ (this work), PSf [72] and PPO [71] at 35 °C and 65 °C for the He/CO_2_ separation and comparison with the Robeson’s upper bound. (**d**) Size-selectivity enhancement, expressed in terms of H_2_/CO_2_ or He/CO_2_ selectivity variation versus H_2_ or He permeability variation, obtained after addition of ZIF-8 to various polymers reported in this work and in the literature: Polybenzimidazole (PBI) [59], Matrimid [58], Torlon^®^ (this work), PPEES [64], and PSf [72] PPO [71].

In Figure 7a,b, we compared the relative permeability variations observed on He and CO_2_ permeability after adding ZIF-8 to three glassy polymers, namely Torlon^®^, PPO, and PSf at 35 °C [71,72]. Variations are similar for all polymers, especially in the case of CO_2_, while for Helium, the polymer experiencing the highest increase is Torlon^®^: this can be attributed to its initial low value of permeability and free volume compared to the other polymers.

In Figure 7c, the data are reported in a He/CO_2_ performance plot and compared against the 2008 Robeson’s upper bound. This picture allows to understand that the properties of composite materials are intermediate between the pure polymer and pure filler ones and to clarify the effect of temperature and filler loading. The maximum loadings of ZIF-8 inspected were 16% in the case of PSf and 45% in the case of PPO, [71] while it was 25 wt % in the case of Torlon^®^ inspected here. The temperature was varied between 35 and 65 °C for all sets of membranes. As it can be seen, in all cases the membranes performances, that occupy quite different regions in the plot, experience a significant permeability enhancement after addition of ZIF-8, which takes them closer to the upper bound. Such behavior is consistent with the properties of pure ZIF-8 membranes, also reported in the plot and taken as an average of different literature values. While PSf and PPO experience an enhancement of selectivity, as their initial selectivity is low compared to ZIF-8, Torlon^®^ suffers a decrease of selectivity. The temperature enhances in all cases the performance by moving the points closer to the upper bound.

Finally, the data were compared with those coming from other sources and involving polymers such as Matrimid^®^, PBI, and PPEES for which the H_2_ and CO_2_ permeability values were measured. In Figure 7d, we highlight the effect of adding ZIF-8 to the size-selectivity features of such membranes: in all cases, the high intrinsic permeability of the filler produces an enhancement of the H_2_ or Helium permeability of the matrix, which is generally monotonous. The effect on the selectivity depends on the initial selectivity of the polymer with respect to the gas couple and it can be detrimental, as in the case of selective polymers such as Matrimid^®^, PBI and Torlon^®^, or beneficial as in the case of the poorly selective PSf, PPO, and PPEES. The enhancement of selectivity is in any event limited to about 1.5, while permeability can increase of factors as high as 10. For this reason, we may conclude that ZIF-8 can be added successfully to glassy polymers with low initial values of permeability where this parameter is more crucial than selectivity to the membrane separation process.

### 3.6. Comparison with Models

**Figure 8:**
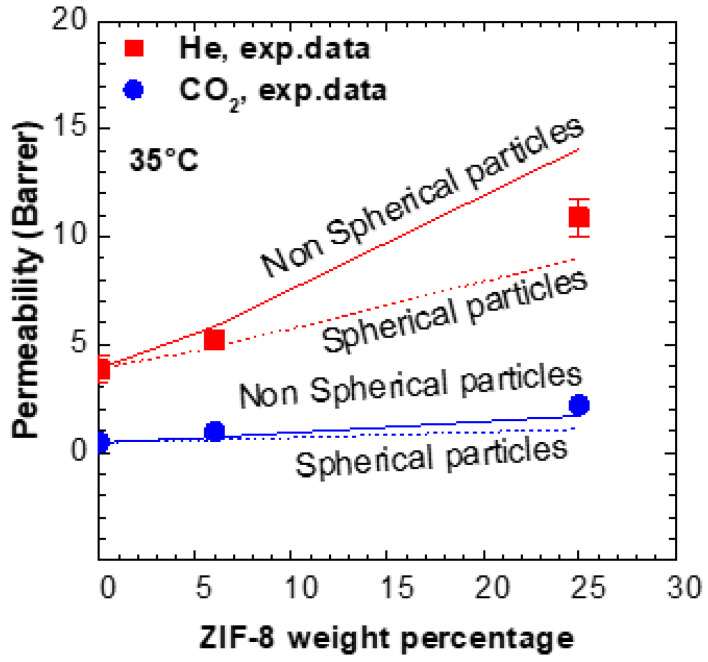
Permeability data in Torlon^®^/ZIF-8 MMMs and predictions of the Maxwell model (spherical particle, *n* = 1/3, dashed line), and Maxwell–Wagner–Sillars model with *n* = 1/6 (non spherical particles, *n* = 1/6, solid line) at 35 °C.

The behavior of the MMMs inspected was compared to a simple model for composites. In particular, we decided to use the Maxwell–Wagner–Sillars model [83], which holds true for composites filled with non-interacting particles below the volume fraction of about 20%. This model was found appropriate in describing the permeability in MMMS formed by polymers and ZIF-8 particles below the recommended loading [71,72]. The generalized equation is reported below:

(5) $${𝒫}_{MMM}=P_{P}\left[\frac{n{𝒫}_{ZIF}+\left(1-n\right){𝒫}_{P}+\left(1-n\right)\left({𝒫}_{ZIF}-{𝒫}_{P}\right)\phi_{ZIF}}{n{𝒫}_{ZIF}+\left(1-n\right){𝒫}_{P}-n\left({𝒫}_{ZIF}-{𝒫}_{P}\right)\phi_{ZIF}}\right]$$

where ${𝒫}_{MMM}$ is the permeability of the mixed matrix membrane, while ${𝒫}_{P}$ and ${𝒫}_{ZIF}$ are those of the pure polymer and the pure ZIF, respectively; $\phi_{ZIF}$ is the volume fraction of the filler and n is the shape factor. When n=1/3, the equation reduces to the Maxwell’s equation and the particles are spherical. For 0≤n≤1/3, the longest axis of the filler is in the same direction of the pressure gradient applied, while for 1/3≤n≤1, we consider oblate ellipsoids, meaning that the shortest axis is in the same direction of the pressure gradient applied. In this work, we decided to compare the data with the model predictions considering the particles spherical (*n* = 1/3, Maxwell’s model, dashed line in Figure 8) and considering a non-spherical factor obtained from data of MMMs based on PPO and ZIF-8 in a previous work (*n* = 1/6, MWS model, solid line in Figure 8) [71]. The Maxwell’s model underestimates the permeability of Torlon/6 ZIF-8 by 7% for Helium, and by 39% for CO_2_. The MWS model, for the same sample, overestimates He permeability by 11% and underestimates CO_2_ permeability by 27%. As the MWS model yields a slightly lower average deviation with respect to the experimental values, it is reasonable to assume that particle of ZIF-8 are weakly non spherical. As expected, both models deviate more significantly from the experimental trend for filler loadings higher than 20%. Overall, the accuracy of the MWS model in representing the MMM behavior is acceptable. The consistency between the model and the data of the MMM indicates that there is a good compatibility and adhesion between the polymer and filler phases.

## 4. Conclusions

We fabricated mixed matrix membranes based on a highly thermal resistant polyamide imide, Torlon^®^, and variable amounts of ZIF-8 up to 25 wt %, with the purpose of enhancing the separation performance of such material in the context of He/CO_2_ and H_2_/CO_2_ separation, especially at increasing temperatures. The effect of filler loading and gas size was analyzed, together with the effect of temperature up to 65 °C. Permeability and diffusivity data were collected and it was found, in particular, that the permeability is a linear decreasing function of the gas kinetic diameter, a correlation which allows to extrapolate the behavior of H_2_.

The results obtained allow to conclude that ZIF-8 is a strong permeability enhancer for Torlon^®^, making its permeability increasing by a factor as high as 3 at 65 °C and with a 25 wt % loading. At the same time, however, the addition of ZIF-8 lowers the He/CO_2_ and H_2_/CO_2_ selectivity of the polymer, by a factor of about 2 for He/CO_2_ in the same conditions, as it is characterized by an intrinsically lower sieving ability towards CO_2_.

The mixed matrix membranes show permeability and selectivity values that are roughly intermediate between the pure polymer and pure filler ones, and follow the models for composite materials in their range of validity, which enables the future design of composite materials with tailored properties. The temperature has a beneficial effect on the He/CO_2_ and H_2_/CO_2_ separation, as it takes the membranes performance closer to the upper bound and can be properly exploited in the separation processes using thermally-resistant membranes, such as the ones inspected in this study. In conclusion, the results of the present work, combined with others involving MMMs formed by polymeric matrices and ZIF-8, suggest that such filler can be used when the separation is controlled by permeability rather than selectivity. For thermally enhanced separations such as the He/CO_2_ and H_2_/CO_2_ ones, the selectivity loss can be reduced by carrying out the separation at a suitable temperature.

## Figures and Tables

**Table 1:** Physical properties of Torlon^®^.

Polymer	Density at 25 °C (g/cm^3^)	T_g_ (°C)	FFV (%)
Torlon^®^ 4000 TF	1.252 [50]	274–276 (DSC, [57]) 281–292 (DMA, [57])	8 [50]

**Table 2:** Physical properties, composition, and reticular structure of ZIF-8.

Filler	Composition [68]	Net [60]	d_a_, Å [34]	d_p_, Å [34]	Surface Area (BET), m^2^/g [68]	Theoretical Density, g/cm^3^	Thermal Stability, °C [68]	Hydrophilicity [68,69]
ZIF-8	Zn(MeIM)_2_	sod	3.4	11.6	1630	0.95 [59] 0.93 [65]	550	Hydrophobic

**Table 3:** Glass transition temperatures measured by DSC.

		T_g_ (°C)
Torlon		267
Torlon/6 ZIF-8		297
Torlon/15 ZIF-8		296

## Supplementary Materials

The following are available online at https://www.mdpi.com/article/10.3390/membranes11120982/s1, Figure S1: Pictures of the membranes produced: Torlon, Torlon/6 ZIF-8, Torlon/25 ZIF-8. Figure S2: SEM images of cross sections of Torlon, Torlon/6 ZIF-8, Torlon/25 ZIF-8 samples. Figure S3: DSC thermograms of the Torlon, Torlon/6 ZIF-8 sample and Torlon/15 ZIF-8 samples. Table S1: MMM samples produced, thickness and tests performed. Table S2: Gas permeability data in Torlon^®^ flat sheet membranes from this work and the literature. Table S3: Gas permeability data in Torlon/ZIF-8 mixed matrix membranes from this work and for ZIF-8 from the literature. Table S4: Ideal selectivity values in Torlon/ZIF-8 mixed matrix membranes from this work and from the literature. Table S5: Gas diffusivity and ideal diffusivity–selectivity in Torlon/ZIF-8 mixed matrix membranes.
