# Supplementary material for: Mixed Matrix Membranes Based on Torlon® and ZIF-8 for High-Temperature, Size-Selective Gas Separations
Source: Membranes (Basel). 2021 Dec 15;11(12):982. doi: 10.3390/membranes11120982 (PMC8703552; doi:10.3390/membranes11120982)
Supplement: Supplementary file 1 [file membranes-11-00982-s001.zip › membranes-1482792-supplementary.pdf]

membranes

Supplementary Materials: Mixed Matrix Membranes Based on Torlon® and ZIF-8 for High-Temperature, Size-Selective Gas Separations

Matilde De Pascale [1,2], Francesco Maria Benedetti [1,3], Elsa Lasseuguette [4], Maria-Chiara Ferrari [4], Kseniya Papchenko [1,4], Micaela Degli Esposti [1,5], Paola Fabbri [1,5] and Maria Grazia De Angelis [1,4,5,*]

1 Department of Civil, Chemical, Environmental and Materials Engineering, University of Bologna, 40131 Bologna, Italy, micaela.degliesposti@unibo.it (M.D.E.); p.fabbri@unibo.it (P.F.)
2 GVS S.p.A via Guido Rossa 30, 40069, Zola Predosa (BO), Italy; matilde.depascale@gvs.it (M.D.P.)
3 Osmoses Inc., 444 Somerville Ave, Somerville, MA 02143, USA, fmben@mit.edu.
4 School of Engineering, University of Edinburgh, Sanderson Building, Robert Stevenson Road, Edinburgh, EH9 3FB, Scotland, UK; K.Papchenko@sms.ed.ac.uk (K.P.); E.Lasseuguette@ed.ac.uk (E.L.); M.Ferrari@ed.ac.uk (M.-C.F.), grazia.deangelis@ed.ac.uk (M.G.D.A.)
5 Italian Consortium for Science and Technology of Materials (INSTM), 50121 Firenze, Italy
* Correspondence: grazia.deangelis@ed.ac.uk

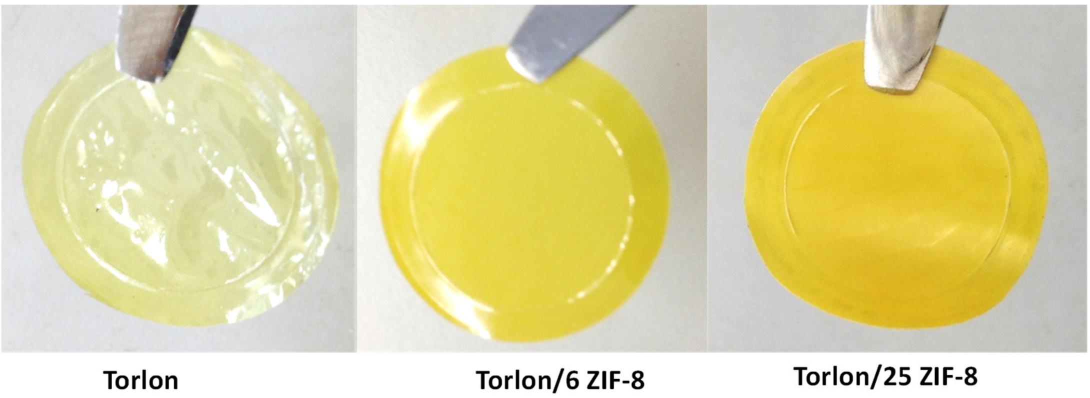

Figure S1. Pictures of the membranes produced: Torlon, Torlon/6 ZIF-8, Torlon/25 ZIF-8.

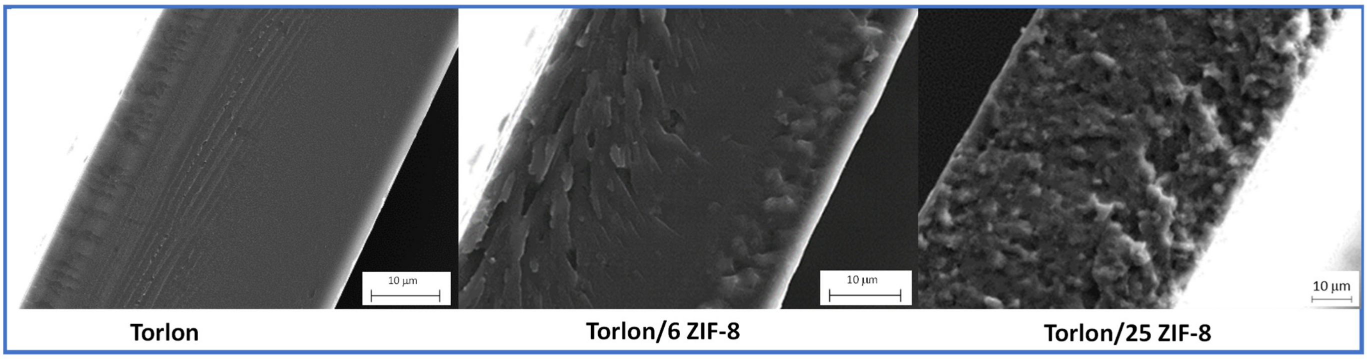

Figure S2. SEM images of cross sections of Torlon, Torlon/6 ZIF-8, Torlon/25 ZIF-8 samples.

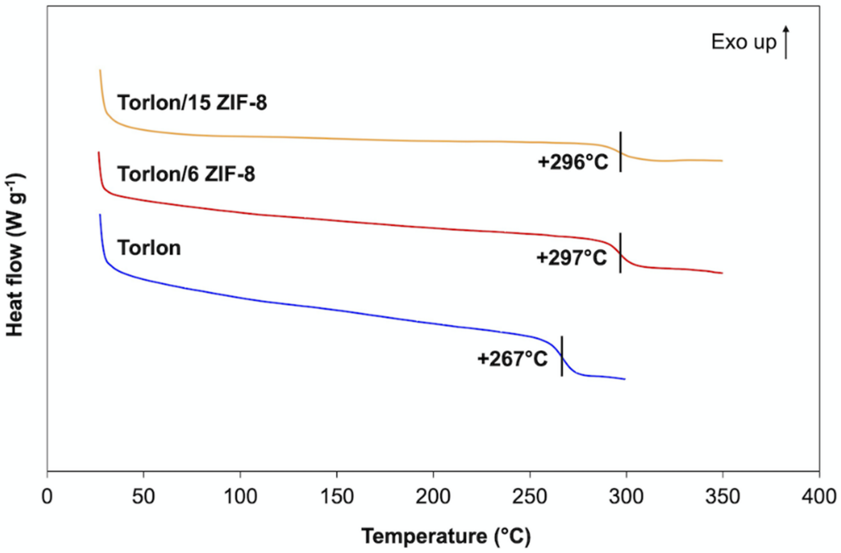

Figure S3. DSC thermograms of Torlon, Torlon/6 ZIF-8 and Torlon/15 ZIF-8 samples.

Table S1. MMM samples produced, thickness and tests performed.

ZIF-8 Weight %	Sample ID	Thickness (μm)	Permeability Tests	SEM Tests	DSC Tests
0	Torlon	10.5±1.4	He, CO_2, 35°C, 65°C	x	x
6	Torlon/6 ZIF-8	45.5±1.9	He, H_2, CO_2, 35°C, 65°C	x	x
15	Torlon/15 ZIF-8	-	-	-	x
25	Torlon/25 ZIF-8	64.7±5.0	He, CO_2, 35°C, 65°C	x	-

Table S2. Gas permeability data in Torlon® flat sheet membranes from this work and the literature.

	Permeability (Barrer)								
Polymer Density	He	H_2	O_2	N_2	CH_4	CO_2	T	Source	Notes
							°C		
1.252	-	3.730	0.130	0.018	0.013	0.541	35	[1]	a
n.r.	5.53	4.44	0.212	0.037	0.030	0.83	35	[2]	b
n.r.	4.4±0.2	-	0.12±0.006	0.014±0.0007	0.009±0.0004	0.47±0.02	35	[3]	c
1.252 [1]	3.9±0.6	-	-	-	-	0.47±0.06	35	This work	d

[a] Torlon 4000 TF, solvent NMP, dense films 50 microns, thermal treatment at 250°C for 12 h, upstream pressure 3.5 atm
[b] Torlon 4000 T, solvent NMP, upstream pressure 10 atm
[c] Torlon 4000T-LV, dense film, solvent NMP, membrane treated at 310°C for 1d
[d] Torlon 4000TF, dense film, solvent NMP, membrane treated at 200°C under vacuum overnight, upstream pressure 1.3 bar, downstream pressure vacuum.

Table S3. Gas permeability data in Torlon/ZIF-8 mixed matrix membranes from this work and for ZIF-8 from the literature. Data marked with an asterisk are estimated values based on linear interpolation between permeability and kinetic diameter in each matrix.

Permeability (Barrer)	He	H_2	CO_2	He	H_2	CO_2
		35°C			65°C	
**Torlon**	3.9±0.6	2.43*	0.47±0.06	6.4±1.0	3.97*	0.72±0.09
Torlon/6 ZIF-8	5.3±0.2	3.8±0.2	0.97±0.04	9.0±0.4	7.0±0.3	1.4±0.1
Torlon/25 ZIF-8	10.0±0.9	6.66*	2.20±0.2	19.6±1.6	13.0*	4.2±0.4
		**20-35°C**				
ZIF-8 [4,5]	3000±1322		1640±712			

Table S4. Ideal selectivity values in Torlon/ZIF-8 mixed matrix membranes from this work and 8 from the literature.

**Ideal Selectivity**	**He/CO_2**	**H_2/CO_2**	**He/CO_2**	**H_2/CO_2**
	**35°C**		**65°C**	
**Torlon**	8.2	5.2*	8.9	5.5*
Torlon/6 ZIF-8	5.4	3.9	6.6	5
Torlon/25 ZIF-8	5.0	3.0*	4.6	3.1*
	**20-35°C**			
ZIF-8 [4,5]	1.8	3.9		

Table S5. Gas diffusivity and ideal diffusivity-selectivity in Torlon/ZIF-8 mixed matrix membranes.

	**Diffusivity (cm^2/s)**				**Diffusivity-Selectivity α_D**	
	**He**	**CO_2**	**He**	**CO_2**	**He/CO_2**	
	**35°C**		**65°C**		**35°C**	**65°C**
Torlon	$(1.6\pm0.4)\times10^{-7}$	$(3.5\pm1)\times10^{-10}$	$(1.6\pm0.4)\times10^{-7}$	$(1.4.\pm0.4)\times10^{-9}$	448	116
Torlon/6 ZIF-8	$(4.4\pm0.4)\times10^{-7}$	$(9.6.\pm0.8)\times10^{-10}$	$(4.5\pm0.4)\times10^{-7}$	$(2.3.\pm0.2)\times10^{-9}$	457	198
Torlon/25 ZIF-8	$(6.6\pm1)\times10^{-7}$	$(2.3\pm0.4)\times10^{-9}$	$(1.3\pm0.2)\times10^{-6}$	$(9.3.\pm1.4)\times10^{-9}$	290	139

References

1. W.F.; Yong, F.Y.; Li, T.S.; Chung, Y.W.; Tong, Molecular interaction, gas transport properties and plasticization behavior of cPIM-1/Torlon blend membranes, J. Memb. Sci. 462 (2014) 119–130. https://doi.org/10.1016/j.memsci.2014.03.046.
2. S.S.; Hosseini, T.S.; Chung, Carbon membranes from blends of PBI and polyimides for N2/CH4 and CO2/CH4 separation and hydrogen purification, J. Memb. Sci. 328 (2009) 174–185. https://doi.org/10.1016/j.memsci.2008.12.005.
3. M.R.; Kosuri, W.J.; Koros, Defect-free asymmetric hollow fiber membranes from Torlon®, a polyamide-imide polymer, for high-pressure CO2 separations, J. Memb. Sci. 320 (2008) 65–72. https://doi.org/10.1016/j.memsci.2008.03.062.
4. H.; Bux, F.; Liang, Y.; Li, J.; Cravillon, M.; Wiebcke, J.; Caro, Zeolitic Imidazolate Framework Membrane with Molecular Sieving Properties by Microwave-Assisted Solvothermal Synthesis, J. Am. Chem. Soc. 131 (2009) 16000–16001. https://doi.org/10.1021/ja907359t.
5. K.S.; Park, Z.; Ni, A.P.; Cote, J.Y.; Choi, R.; Huang, F.J. Uribe-Romo, H.K.; Chae, M. O'Keeffe, O.M.; Yaghi, Exceptional chemical and thermal stability of zeolitic imidazolate frameworks, Proc. Natl. Acad. Sci. 103 (2006) 10186–10191. https://doi.org/10.1073/pnas.0602439103.
6. Q.; Song, S.K.; Nataraj, M. V.; Roussenova, J.C.; Tan, D.J.; Hughes, W.; Li, P.; Bourgoin, M.A.; Alam, A.K.; Cheetham, S.A. Al-Muhtaseb, E.; Sivaniah, Zeolitic imidazolate framework (ZIF-8) based polymer nanocomposite membranes for gas separation, Energy Environ. Sci. 5 (2012) 8359. https://doi.org/10.1039/c2ee21996d.
